# Effects of carbohydrate drinks ingestion on executive function in athletes: a systematic review and meta-analysis

**DOI:** 10.3389/fpsyg.2023.1183460

**Published:** 2023-08-10

**Authors:** Jingye Yang, Qi Han, Qi Liu, Tieying Li, Yongcong Shao, Xuemei Sui, Qirong Wang

**Affiliations:** ^1^College of Exercise Science, Beijing Sport University, Beijing, China; ^2^Sports Nutrition Center, National Institute of Sports Medicine, Beijing, China; ^3^Key Lab of Sports Nutrition, State General Administration of Sport of China, Beijing, China; ^4^School of Psychology, Beijing Sport University, Beijing, China; ^5^Arnold School of Public Health, Department of Exercise Science, University of South Carolina, Columbia, SC, United States

**Keywords:** cognition, executive function, carbohydrates, sugars, athletes, exercise

## Abstract

**Background:**

Carbohydrates are often used as boosters for endurance and high-intensity exercise. However, it is unclear whether carbohydrate drinks intake before or during exercise can affect specific domains of cognitive function, such as Executive Function (EF).

**Methods:**

Following the guidance of PRISMA 2020, we searched six major databases including PubMed, WOS, SPORTDiscus, Cochrane, Embase, and Scopus. Outcomes were presented in the form of Reaction Time (RT), Accuracy (ACC), and Scores (Score) for performing EF tests. Effect sizes were calculated from the test results of EF and expressed as standardized mean differences (SMDs). After analyzing the overall results, we performed subgroup analyses based on the athletes’ program characteristics.

**Results:**

After retrieving a total of 5,355 articles, ten randomized controlled trials (RCTs) were identified and included in this review. The overall results showed that the intake of carbohydrate drinks before or during exercise did not have a significant effect on the reduction of EF after exercise (ACC (−0.05 [−0.27, 0.18]); RT (−0.18 [−0.45, 0.09]); Score (0.24 [−0.20, 0.68])). The subgroup analyses based on open skill sports and close skill sports also showed invalid results, but the results of RT ended up with different preference (ACC of open skill sports athletes (−0.10 [−0.34, 0.14]); RT of open skill athletes (−0.27 [−0.60, 0.07]); RT of close skill athletes (0.29 [−0.24, 0.82])).

**Conclusion:**

The intake of 6–12% of single or mixed carbohydrates before or during exercise was not significantly effective in reducing the decline in EF after exercise. Our findings may have been influenced by the type of intervention, dose, mode of administration, or individual variability of the included subjects.

## 1. Introduction

Cognition is the mental action or process of acquiring knowledge and understanding through thought, experience, and the senses (Sadkhan, 2018). In psychology, cognition is often divided into several domains, which are not independent of each other (Harvey, 2022). In the sports field, cognitive functions, which are frequently measured, include Executive Function (EF), Information Processing, Spatial Ability, Attention, and Memory (Yongtawee et al., 2021). Executive Function, the focus of this study, refers to putting thought ahead of action, promoting mastery of new challenges, and helping to maintain focus during periods of sensory overload (Koch and Krenn, 2021), which plays a vital role in sports. For example, athletes with better EF may achieve higher levels of athletic performance than other athletes (Voss et al., 2010). For high-level athletes, EF could vary depending on the sport and be especially important in strategic sports (Krenn et al., 2018). For example, regional volleyball players are better than provincial players both in volleyball-specific skills and general cognitive functioning (such as EF), which can be used to determine athletes of different competitive levels (Formenti et al., 2022). A study also suggested that EF was important enough to predict the future success of soccer players (Vestberg et al., 2012). And training targeting speed, agility and quickness and cognitive engagement should be used into soccer training programs as a useful strategy to promote physical fitness and cognitive domains in soccer players (Trecroci et al., 2022). Due to the highest level of sports performance requires a host of cognitive functions (Walton et al., 2018), identifying ways to reduce cognitive decline during exercise is crucial. Sports drinks (carbohydrate-electrolyte replacement drinks) are well known dietary supplements and have been shown to improve performance in endurance and ultra-endurance sports to promote hydration and provide additional carbohydrate substrate to glycogen-depleted muscles (Burke and Read, 1993). However, it is unclear whether carbohydrate supplementation before exercise is beneficial for cognitive function (Baker et al., 2014). It is also unclear whether carbohydrate ingestion affects specific cognitive domains, such as EF, after exercise.

Carbohydrates (CHO) are often used as an ergogenic aid for both endurance and high-intensity sports (Cermak and van Loon, 2013). Previous review has shown that carbohydrate supplementation appears to improve cognitive performance in soldiers engaged in sustained, high-intensity physical activity that consumes high levels of energy (Lieberman, 2003). A study also showed that intake of no more than 8%, 30–80 g/h of CHO can enhance exercise performance during endurance exercise ≥1 h (Temesi et al., 2011). The primary mechanism of carbohydrate supplementation for this enhanced performance is through high CHO delivery rates (>90 g/h), resulting in high CHO oxidation rates (Stellingwerff and Cox, 2014). In competition or training status, fatigue can lead to a decrease in an athlete’s ability to continue to perform at the highest level (Rahnama et al., 2003). Fatigue can be subdivided into physical and/or mental fatigue symptoms, each with a unique but ultimately complex, multifactorial, and heterogeneous pathophysiology (Twomey et al., 2017). Mental fatigue is a psychobiological state caused by prolonged and demanding cognitive activity (Van Cutsem et al., 2017), and evidence suggests a strong link between cognitive function and mental fatigue (Ishii et al., 2014). A review has suggested that brain carbohydrate metabolism may be an important factor influencing endurance exercise fatigue (Meeusen, 2014). And it has also been provided the factor inducing central fatigue might be hypoglycemia because of brain glycogen reduction during prolonged exercise (Matsui et al., 2011). So in recent years, more studies have begun to examine the impact of nutritional interventions, including carbohydrate drinks, on cognition in sports (Meeusen, 2014). In a study of badminton players, the authors concluded that there was no significant effect of CHO on reaction time on the Stroop test, and recommended further research to prove that cognitive function may be a potential mechanism for improving badminton serve accuracy after carbohydrate intake (Bottoms et al., 2012). Another study on intermittent exercise indicated that carbohydrate intake in more complex and cognitively challenging situations did not significantly improve cognitive function (Welsh et al., 2002). In contrast, a study that examined high-intensity intermittent exercise found that CHO intake, regardless of type, improved cognitive performance throughout exercise, especially in difficult cognitive tasks (Dupuy and Tremblay, 2019). The results of a study on squash players suggested that CHO intake and possibly associated elevated blood glucose concentrations may improve cognitive function (Bottoms et al., 2006). Based on that cognition plays a key role in sports performance, and the data on the effects of CHO intake on cognitive function, particularly EF, are unclear, so additional research is needed.

To sum up, owing to maintaining the stage of EF of athletes is essential during competition and frequently using of carbohydrate drinks in athletes, it’s meaningful to figure out the impact of carbohydrate drinks on EF of athletes, which may provide ways to improve sports performance in the future. In a recent meta-analysis (Oliver et al., 2021), the authors introduced the effects of different nutritional interventions, including carbohydrates, on accuracy and reaction time in post-exercise cognitive function, but this article did not indicate the corresponding subdomains of cognitive function. Moreover, the literature they reviewed included carbohydrate supplementation in the form of mouth-rinsing, which was shown to stimulate reward centers in the brain and increase corticomotor excitability (Rollo and Williams, 2011). A further review suggests that recent advances in understanding the regulation of energy supply (primarily glucose) for neuronal function suggest that it is too simplistic to assume that carbohydrate intake will inevitably improve cognitive function (Gibson, 2007). Due to the different conclusions obtained and the fact that most previous studies did not examine specific subfields of cognitive function, the purpose of this meta-analysis will focus on one of the most important cognitive domains of athletes, EF, to explore whether carbohydrate intake in the form of sports drink, has an effect on EF in athletes.

## 2. Methods

This systematic review and meta-analysis were conducted under the Preferred Reporting Items for Systematic Reviews and Meta-analyses (PRISMA) 2020 statement (Page et al., 2021).

### 2.1. Registration

This article has been registered on Prospero in advance, and the registration ID is CRD42022377215.

### 2.2. Eligibility criteria

The eligibility criteria for inclusion in the study followed the PICOS model: population, intervention, comparator, outcome, and study design. The population was limited to healthy athletes, professional or amateur, and people who were actively involved in sports. The intervention was the consumption of sports drinks containing 6–12% single or complex carbohydrates before or during exercise. The comparators were the placebo or distilled water. Outcome indicators are all test results related to EF, including cool EFs and hot EFs. However, due to the limited number of articles included, the final literature included only results on cool EFs. The studies included were randomized controlled trials on humans, including single-blind or double-blind designs. Crossover trails and parallel group design studies were all included.

EF is often divided into the ordinary level and advanced level, where the ordinary level includes working memory, inhibitory control, and cognitive flexibility, while the advanced level includes reasoning, problem-solving, and planning (Diamond, 2013). Regarding working memory, it is often measured by Complex Span (Cspan) tasks, Updating tasks, Recall N-back (RNb) tasks, and Binding tasks (Wilhelm et al., 2013). The paradigm for the subfield of test inhibition typically includes the Stroop task, Simon task, Flanker task, antisaccade tasks, delay-of-gratification tasks, go/no-go tasks, and stop-signal tasks (Diamond, 2013). Tests commonly used to measure cognitive flexibility include the Wisconsin Card Sorting Test and classic fluency tasks which can be classified as either verbal fluency, design fluency, or tests of divergent thinking (Rende, 2000). Our inclusion results were filtered from the results of all tests of EF.

The following studies were excluded based on PICOS principle. The population who was not healthy athletes, professional or amateur, and people who were not actively involved in sports, such as animal studies, experiments that focused on non-healthy or injured participants. Interventions in the form of mouthwash or nasal spray, non-liquid supplements or did not involve the ingestion of sports drinks. The comparators without the placebo or distilled water. The outcome indicators without the results of all tests related to EF. The studies that are not randomized controlled trials on humans, or non-randomized controlled trials. The articles with full text are not available or reviews.

### 2.3. Information sources

We searched six English databases including PubMed, Web of Science (WOS), SPORTDiscus, Cochrane, Embase, and Scopus. The last search date was February 9th, 2023. The final results included 5,355 articles.

### 2.4. Search strategy

We searched the Medical Subject Headings (MeSH) Database for Carbohydrates, Sugars, Glucose, Beverages, Sugar-Sweetened Beverages, Athletes, Sports, Exercise, Cognition, and Central Nervous System. Use all these MeSH terms and Entry terms for the six database searches. Specific search formulas are included in File 1 of Supplementary materials.

### 2.5. Selection process

The screening of articles was performed through three stages. After removing duplicates, the initial screening was done based on the article titles, and articles that were completely irrelevant were eliminated. The second stage excluded articles that did not meet the requirements, based on reviewing the abstract of the article. In the third stage, the researchers read all the remaining articles in full and decided which ones should be included based on the inclusion and exclusion criteria. This process was completed by two of the authors, JY and QL, and when there was disagreement, they discussed the study in question or asked a third author for input until a consensus was reached.

### 2.6. Data collection process

First, all data from the articles were extracted by JY. After the extraction was completed, QH checked and verified the data until they agreed on the results. All data were extracted from the tables and images in the article. The data in the tables was extracted directly. In the case of data displayed only in graphical format, zoom in on the image to improve the accuracy of data estimation and extract the data using Webplotdigitizer: Version 4.6 (Rohatgi, September, 2022).

### 2.7. Data items

We extracted the following information from the selected articles: basic information about the study (authors and publication years), research design, subject characteristics (sample size, sex, age, and types of sports programs engaged in), types and concentrations of supplements, timing and frequency of supplementation, exercise protocol, and psychological paradigms.

Then, any data relevant to the EF test results were extracted from these eligible articles. The final results included Reaction Time (RT), Accuracy (ACC), and scores of EF (Score). RT is defined as the time interval between the appearance of the stimulus and the appearance of an appropriate voluntary response in the subject (Jain et al., 2015). The ACC is a measure of performance on a task, usually defined as the proportion of correct responses. Score reflects an individual’s ability to resist cognitive interference and this measurement was only used in one article (Winnick et al., 2005).

### 2.8. Study risk of bias assessment

A revised Cochrane risk-of-bias tool, RoB 2 tool (Higgins et al., 2016), was used for assessing the risk of bias in randomized trials. RoB2 has scales specifically for crossover and parallel experiments to more accurately assess the risk of bias. There are five domains included in RoB2: Bias arising from the randomization process, Bias due to deviations from intended interventions, Bias due to missing outcome data, Bias in the measurement of the outcome, and Bias in the selection of the reported result. For crossover experiments only, the RoB 2 tool has a dedicated evaluation field, domain S, which includes analysis of bias arising from period and carryover. The risk of bias in each area is categorized as “low risk,” “some concern,” and “high risk. Please refer to the official Cochrane website, https://methods.cochrane.org/risk-bias-2, for a description of each subfield. The process was done by JY and QL, and when there was disagreement, the two discussed or asked QH for help until a consensus was reached.

### 2.9. Synthesis methods

Since the data processed were continuous data such as RT, ACC, or Score, we chose to use the standardized mean differences (SMDs) to measure the magnitude of the effect sizes (Takeshima et al., 2014). Otherwise, the vast majority of the included studies did not present data for both phases or each subject. Only the Means and Standard Deviations (SD) or Standard Error (SE) of the combined data for the two phases of the experimental and control groups were provided in the articles. Because it has been demonstrated that interpolation of missing standard deviations in the meta-analysis can provide accurate results (Furukawa et al., 2006), we finally chose to approximate a paired analysis by imputing missing standard deviations.

Each set of experiments contains one baseline measurement and one post-intervention test. We used SD_change_ to denote the standard deviation of the mean change from the baseline. The calculation formula [Eq. (1)] is shown below (Yagiz et al., 2022). In order to calculate SD_change_, a correlation *r* between baseline and post-intervention levels is also required, and this information is usually not available from trial reports and must be estimated (Furukawa et al., 2006). It has been shown that estimating an *r* of 0.5 yielded a value closer to the actually reported value (Balk et al., 2012), so in this study, we choose a correlation of 0.5 for the estimation of the SD_change_ of crossover trails. For a parallel trial, the value of *r* is always 0. In total, nine of the included studies were crossover experiments and one was a parallel experiment. In four of these studies, the experiments provided SE but not SD, so we used the following Eq. (2) to converted SE to SD (Elbourne et al., 2002) before calculations were performed. After calculating the standard deviation of the differences, we entered the obtained values directly into Review Manager 5.4.1 ((RevMan), 2020) and performed the calculation.

RevMan has embedded algorithms to perform effect size calculations for different data types (Deeks and Higgins, 2010). It automatically uses the Hedges’ (adjusted) g method, which is considered more suitable for calculating effect sizes for small samples (<20) (Durlak, 2009). The interpretation of the effect size follows Cohen’s recommendations (Cohen, 2013): small effect (indiscernible to the naked eye) = 0.2, medium effect = 0.5, large effect (visible to the naked eye) = 0.8.

Heterogeneity was performed by *I*^2^ statistic. The results are described as low, medium, and high and are assigned to the *I*^2^ values of 25, 50, and 75% (Higgins et al., 2003). Due to the differences between the subjects we chose to study and the doses of the interventions, we chose a random-effects model to calculate the effect sizes (Borenstein et al., 2010), and the confidence interval was set at 95%. Finally, a sensitivity analysis was performed by RevMan and articles with high effect on the results were excluded.


(1) $$\mathrm{SDchange}=\sqrt{{\mathrm{SD}}^{2}\mathrm{pre}+{\mathrm{SD}}^{2}\mathrm{post}-\left(2\mathrm{rSDpreSDpost}\right)}\;$$

(2) $$\mathrm{SE}=\frac{\mathrm{SD}}{\sqrt{n}}\;$$

## 3. Results

### 3.1. Study selection

**Figure 1:**
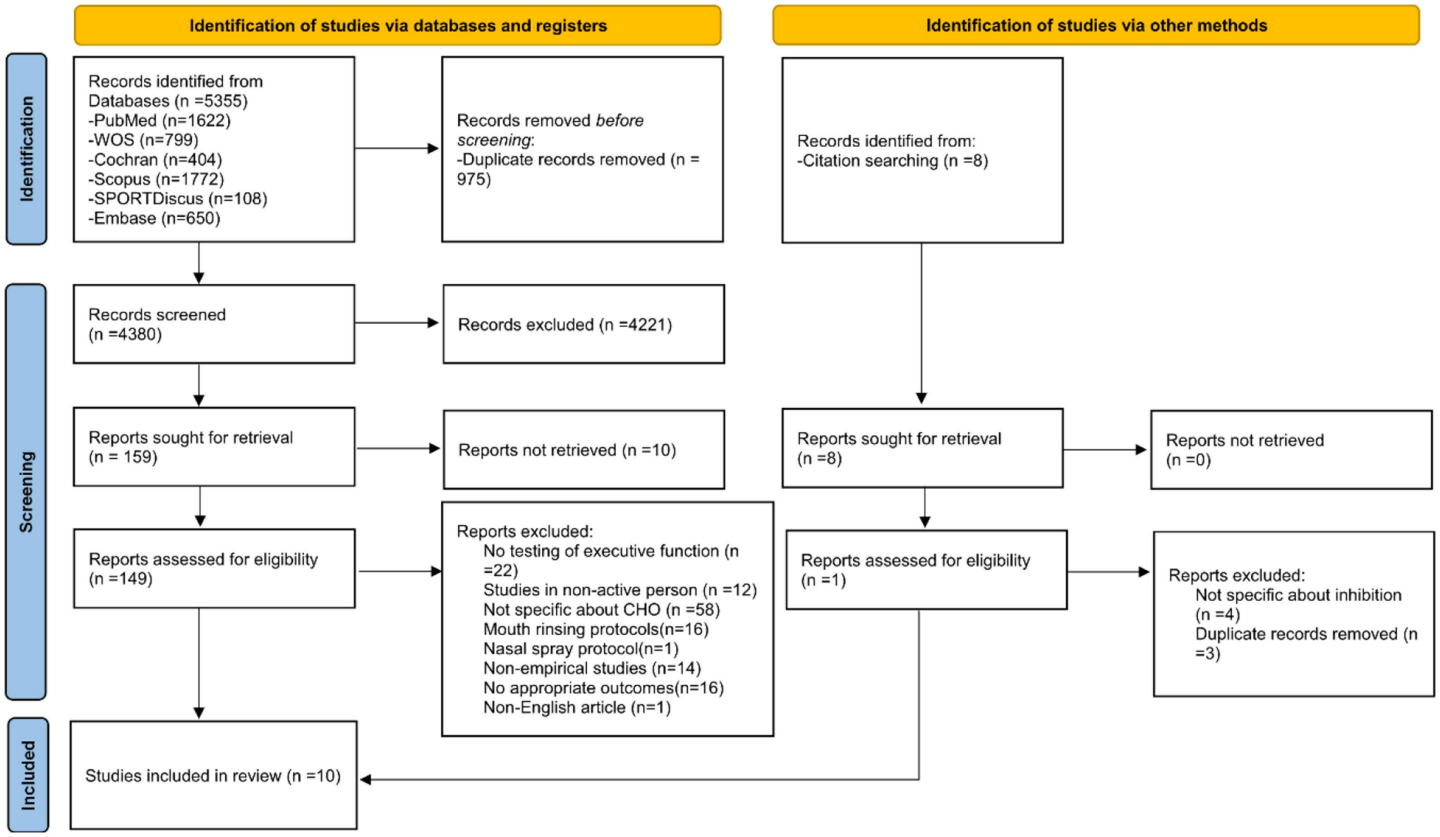
Flow diagram of study selection according to the PRISMA 2020 statement.

A search of six major databases resulted in 5355 articles, and another 8 articles were found from the citations in those articles. Among the articles, there were 975 duplicate records. After these duplicate records were removed, a total of 4,380 articles were screened and 149 of these articles were assessed. Of the eight cited articles, seven were excluded and one was included. Finally, a total of 10 RCTs were included which encompassed both crossover trials and parallel trials. The complete flow diagram was shown in Figure 1.

### 3.2. Study characteristics

Ten RCTs were included, involving nine crossover experiments (Hogervorst et al., 1999; Winnick et al., 2005; Wong et al., 2014; Pollow et al., 2016; Pomportes et al., 2016; Harper et al., 2017; Pomportes et al., 2019; Sun et al., 2020; Zhu et al., 2020), and one parallel experiment (Dupuy and Tremblay, 2019). The details of each study are listed in Table 1.

**Table 1:** Study characteristics.

Study	Experimental design	Subjects	Supplementation	Fluid replenishment time and dose	Exercise protocol	Results
Hogervorst et al. (1999)	Double-blind RCT with a crossover design	15 healthy male competitive cyclists or triathletes (23.3 ± 3.6 years)	1) Placebo water 2) Placebo- carbohydrates 68.8 g/L 3) carbohydrates combined with caffeine 150 mg/L 4) with caffeine 225 mg/L 5) with caffeine 320 mg/L. Each subject was tested at different times for 5 solutions.	With 8 mL fluid/kg body weigh ingestion before trial at warm-up session and 3 mL fluid/kg body weigh ingestion every 20 min during the trial.	An all-out 1 h time trial on a bicycle ergometer.	The Stroop Color-Word Test. Signal Detection Task. The Motor Choice Reaction Time Test and the visual Verbal Learning Test with Interference were measured.
Pomportes et al. (2019)	Single-blind RCT with a pseudo counter-balanced design	10 pentathlon athletes (6 males and 4 females, age 18.6 ± 2 years)	1) A 6% carbohydrate complex 2) A 200 milligrams (mg) caffeine added with orange sugarless syrup 3) A 3.4 grams (g) guarana complex 4) A placebo.	Two before exercise and one during exercise.	The exercise involved a 40 min run on a treadmill at a steady speed and each athlete participates four times.	Simon tasks were measured.
Winnick et al. (2005)	Double-blind RCT	20 active men (*N* = 10,24.9 ± 2.08 years) and women (*N* = 10,22.9 ± 1.45 years), with experience competing in team sports.	1) A 6% CHO solution 2) A flavored placebo (PBO).	Subjects received 5 mL/kg of fluid before exercise and 3 mL/kg after exercise, in addition to 3 mL/kg over a 5 min span after the first and third quarters, and 8 mL/kg during a 20 min halftime. Consumption of carbohydrate was approximately 41 g h^−1^ in the CHO trial.	Four 15 min quarters of shuttle running with variable intensities ranging from walking (30%VO_2_max), to running (120%VO_2_max), to maximal sprinting, and 40 jumps at a target hanging at 80% of their maximum vertical jump height.	The Stroop Color and Word Test were measured.
Zhu et al. (2020)	A single-blind RCT with a cross-over design.	14 male soccer athletes (age, 24.3 ± 3.7 years)	1) Control (electrolyte solution without CHO) 2) CHO (4.2 g/100 mL CHO, 3 mL/kg in each trial), 3) CHO-M (brief MBI).	Participants were instructed to consume 3 mL/kg in each trial during the half-time break.	Soccer games.	The Stroop effect task (ST), Corsi-block-tapping test (CBT), and rapid visual information processing task (RVIPT)
Dupuy and Tremblay (2019)	RCT	85 recreationally active males (24.4 ± 4.6)	1) Water (control) 2) Glucose (60 g/L) 3) A commercial sports drink (CSD) 4) Diluted maple syrup 5) Concentrated maple water.	Subjects ingested 166 mL of the experimental solution, drinking a total of 1 L of the same solution throughout the experimentation.	Six 3 min bouts at 95% of their maximal aerobic power on a stationary bike, with 3 min of passive rest between efforts.	Plasma glucose concentration, peak power output, maximal oxygen consumption, cognitive task and cerebral oxygenation were measured. Cognitive performance was assessed by the computerized modified Stroop task.
Harper et al. (2017)	Randomised, crossover.	15 male University soccer players (age: 22 ± 2 years)	1) Water; WAT 2) Carbohydrate-electrolyte, 12% CHO, 60 g·500 mL^−1^ 3) Placebo-electrolyte; PL)	Beverages were ingested towards the end of the warm-up (250 mL) and at HT (250 mL); both <15 min before each half commenced.	90 min of soccer-specific exercise (including self-paced 6 exercise at the end of each half).	Physical, technical and cognitive (memory, attention, decision making) performance were assessed.
Sun et al. (2020)	Double-blind RCT with a crossover design	16 male college soccer players (21 ± 1 years)	1) Carbohydrate-electrolyte-solution (CES,6% CHO in the form of sucrose) 2) Carbohydrate-electrolyte-protein-solution (CEPS,4% CHO in the form of sucrose plus 2% whey PRO) 3) Placebo (PLA).	Before warming up (5 mL/kg BM) and every 15 min thereafter (during resting period, 2 mL/kg BM).	In each trial, they completed 30 min of 70% VO_2_peak cycling, and one of two solutions (CES or CEPS) was consumed.	The Visual Search Test, the Stroop Test, and the Rapid Visual Information Processing Test were measured.
Pomportes et al. (2016)	A double blind, randomized, crossover research design	17 high-level squash and fencing athletes (7 females and 10 males, 19.1 ± 1 years)	1) 7% carbohydrate complex (CHO: fructose and maltodextrine, Isoxan Sport) 2) Placebo (Pl, 250 mL tap water added with orange sugarless syrup).	During each experimental session, subjects ingested 60 min (Ing1) and 30 min (Ing2) before exercise either 250 mL of a 7% carbohydrate complex or placebo.	6 sprints (5 s) with a passive recovery (25 s) followed by 15 min submaximal cycling.	Physiological parameters, perceived exertion, shooting performance and cognitive function were measured. Cognitive function included a simple reaction time (SRT) task at rest, a visual scanning task (VS) and a Go/No-go task (GNG) during a submaximal cycling exercise.
Wong et al. (2014)	Randomized crossover study design	9 healthy active males (*n* = 9) and 10 eumenorrheic females (*n* = 10), 23.1 ± 1.2 years for males, 22.6 ± 0.8 years for females.	1) Carbohydrate-electrolyte solution (CES, CHO 6.6%) 2) Lemon tea (LT) 3) Distilled water (DW)	The total intake volume of fluid during REC was equivalent to 150% of BM loss during the previous 60-min run. The fluid was consumed in 6 equal volumes at 30, 60, 90, 120, 150, and 180 min of the REC.	All participants completed a 60 min moderate endurance run at 60% VO_2_max on a motorized treadmill in a hot and humid environment (temperature: 29.2 ± 0.9°C; relative humidity: 71 ± 5%) of 3 times. Immediately after the run, the 4 h recovery (REC) period commenced.	Plasma volume (PV) changes, serum osmolality, plasma sodium, potassium concentrations, abdominal discomfort (AD), stomach fullness (SF), and the CogState battery were measured.
Pollow et al. (2016)	Double-blind RCT with a crossover design.	7 well-trained cyclists and triathletes (26.9 ± 3.9 years)	1) A carbohydrate–electrolyte beverage (5 mL/kg, 6.3% CHO, 18 mM sodium) 2) A capsule containing 6 mg/kg of caffeine 3) A capsule containing a placebo.	A first CHO beverage was consumed 1 h before trials and a second at the onset of each trial.	Two trials to exhaustion (TTE) at 90% VO_2_ peak and two 50 km time trials (TTD)	Exercise performance, metabolic parameters, alertness, cognitive performance and perception of trial substance were measured. Cognitive function included a computerized ANAM^®^ test and Stroop word-color test.

### 3.3. Risk of bias in studies

**Figure 2:**
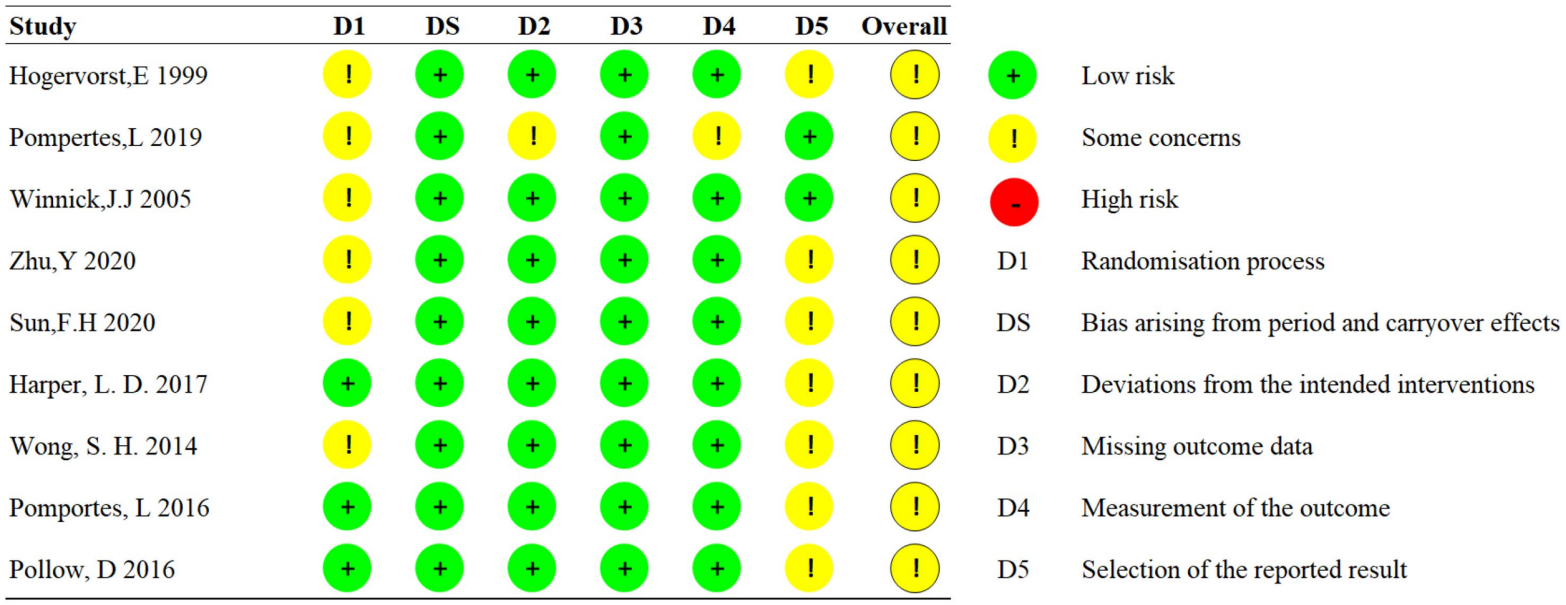
Risk of bias of crossover trails.

**Figure 3:**
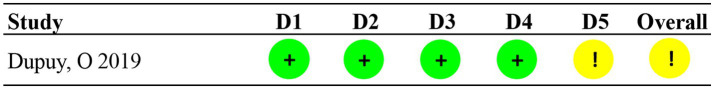
Risk of bias of parallel trail.

**Figure 4:**
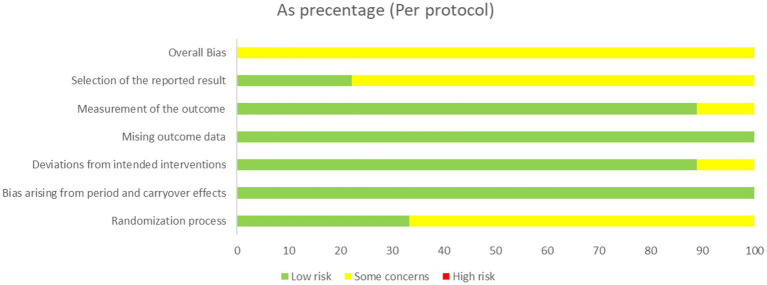
Risk of bias graph of crossover trails.

**Figure 5:**
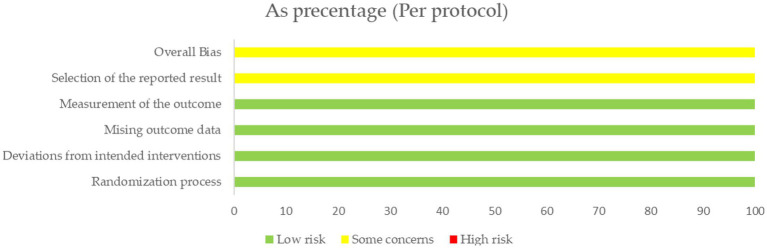
Risk of bias graph of the parallel trail.

Ten crossover trials used the crossover version of RoB2 tool to assess the risk of the outcome. One parallel study used the normal version of the RoB2 tool. The assessment of bias risk for each trial is shown in Figures 2–5. The overall results show that all the literature were presented with some concerns, with the main reasons appearing in the D1 and D5 domains. Namely, the randomization process of D1 and selection of reported results of D5.

### 3.4. Results of individual studies

The required means and standard deviations were calculated from the data of pre- and post-test in our included studies and the formula provided in the data synthesis and brought into RevMan to obtain the following results.

The *I*^2^ of the heterogeneity test were all less than 50%. No articles with abnormal results were found in the sensitivity test. All of the test results were presented in three ways: ACC, RT, and Score. Group analyses were done based on the athletes’ sport type, including open-skill and closed-skill sports.

#### 3.4.1. All results of EF

##### 3.4.1.1. ACC

**Figure 6:**
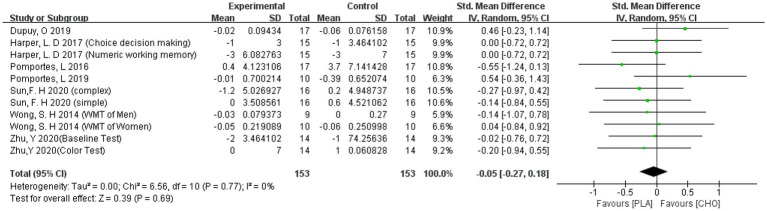
A meta-analysis of ACC of all studies.

The calculated results showed that the carbohydrate drink supplementation did not have a significant effect on the ACC in the EF measurements (−0.05 [−0.27, 0.18]). The results are shown in Figure 6.

##### 3.4.1.2. RT

**Figure 7:**
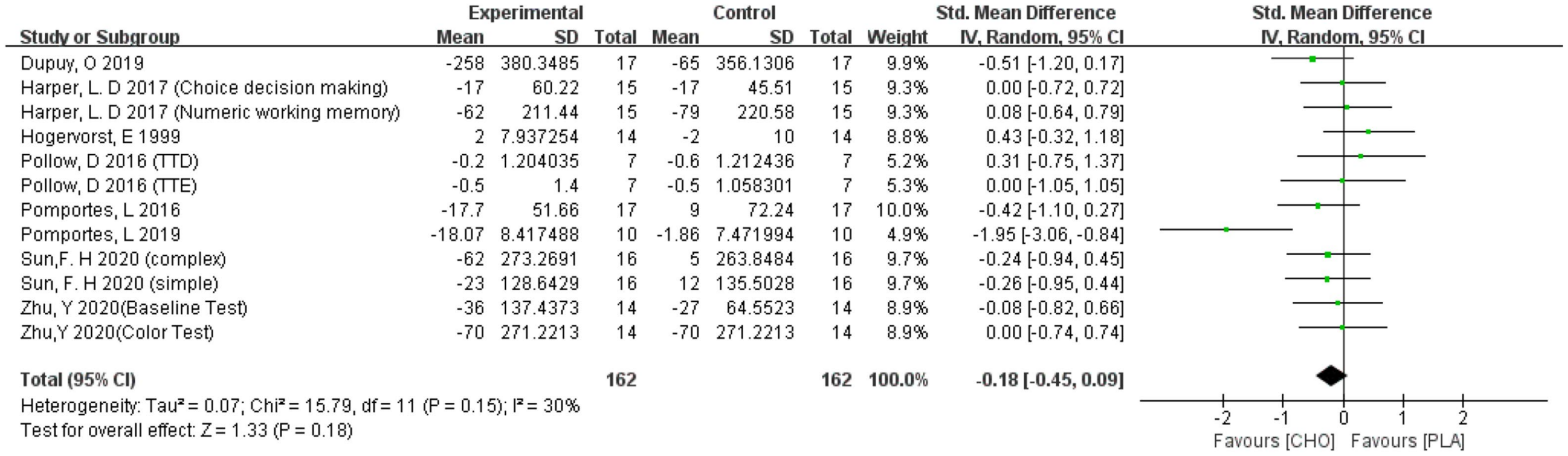
A meta-analysis of RT of all studies.

Pooled results showed that carbohydrate drink ingestion did not have a significant effect on the RT of EF (−0.18 [−0.45, 0.09]). The results are shown in Figure 7.

##### 3.4.1.3. Score

**Figure 8:**
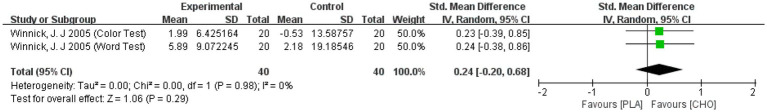
A meta-analysis of the score of all studies.

The final results showed that the carbohydrate drink also had no effect on the scores in the cognitive function test (0.24 [−0.20, 0.68]). The results are shown in Figure 8.

#### 3.4.2. Subgroup analysis

##### 3.4.2.1. ACC of open skill sports athletes

**Figure 9:**
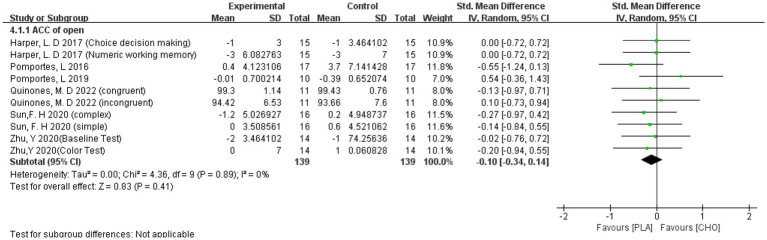
A meta-analysis of ACC of open skill sports athletes.

When only studies in which the athletes were engaged in an open-skill program were included in the ACC (−0.10 [−0.34, 0.14]), the intake of carbohydrate drink did not have an effect on cognitive function after exercise. The results are shown in Figure 9.

##### 3.4.2.2. ACC of closed skill sports athletes

**Figure 10:**
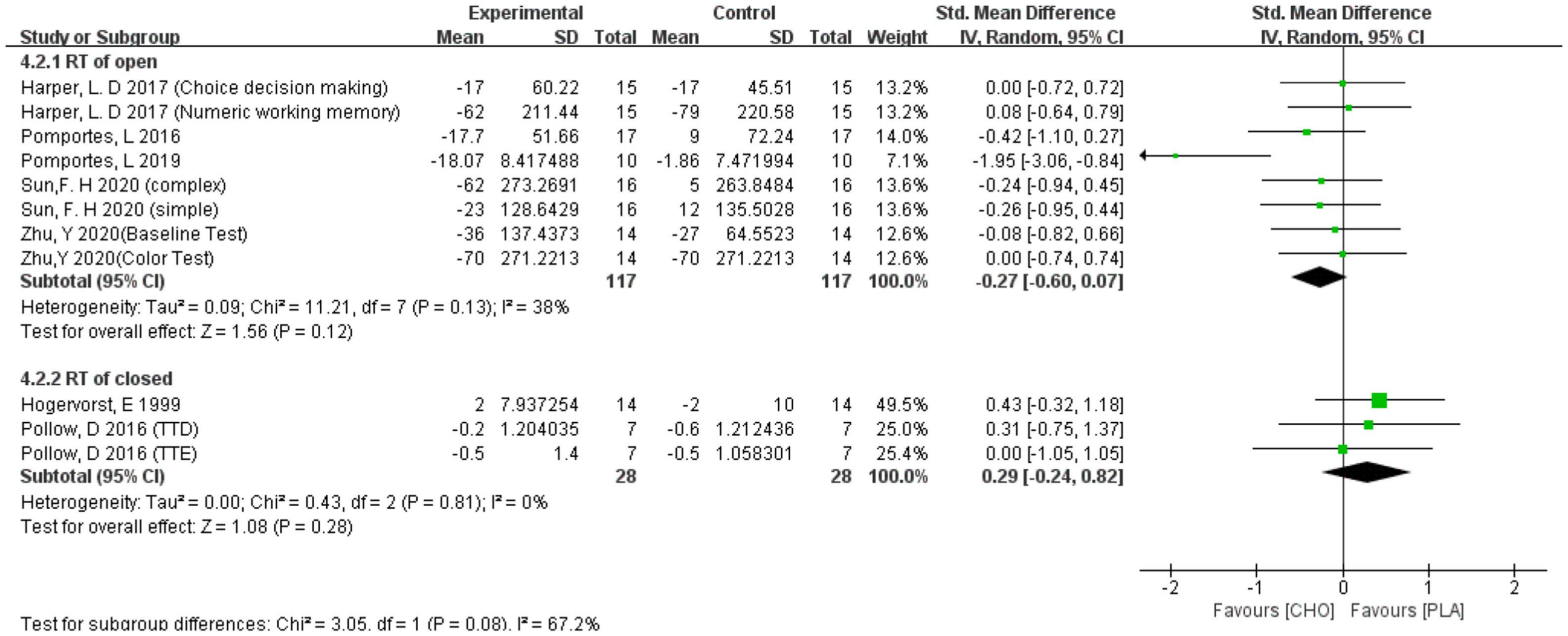
A meta-analysis of RT of open vs. closed skill sports athletes.

Although the final results showed no significant effect of carbohydrate drink intake on either open-skill (−0.27 [−0.60, 0.07]) and closed-skill (0.29 [−0.24, 0.82]) athletes. Although the all results are both invalid, athletes with open skills are more inclined to have effects on EF after taking carbohydrates, while the opposite is true for athletes with closed skills. The results are shown in Figure 10.

## 4. Discussion

Previous studies had shown inconsistent results regarding the effect of carbohydrate intake on cognitive function (Baker et al., 2014), which suggested that the discussion of cognitive function was too broad. The purpose of this literature review was to examine whether carbohydrate drink ingestion before or during exercise has an effect on post-exercise EF.

### 4.1. Explanation of the results

#### 4.1.1. Outcomes of EF of all studies

The data showed that sports drink intake did not have a significant effect on EF. A study suggested that high-intensity exercise, aerobic exercise, and the cognitive demands associated with skill, attention, and decision-making all rely on carbohydrates as an important substrate for energy production (Baker et al., 2015), so why does this lead to invalid results? In the studies we included, the interventions were 6–12% monosaccharide or carbohydrate complex solutions. It has been suggested that for exercise lasting approximately 1 h and possibly for intermittent exercise (sometimes lasting longer than 1 h), athletes consuming a single type of carbohydrate should consume approximately 60–70 g/h for optimal carbohydrate delivery (Jeukendrup, 2007). Among the articles we included, some studies did not meet this standard. For example, in Winnick et al. (2005), the intake rate of CHO was only 41 g h^−1^, and underdosing might be one of the reasons for the invalid results of Score. Second, the type of carbohydrate might also have influenced the results, since the rate of absorption of single carbohydrates in the gut is limited (Jeukendrup, 2008). In one articles (Quinones and Lemon, 2022), the authors investigated the effect of modified corn starch and equivalent amounts of simple sugars on cognitive function in athletes and concluded that the group consuming the modified starch had better cognitive function. In addition, the form of carbohydrate intake also plays a vital role in the impact on cognitive function. In addition to liquid forms, energy gels (Scott et al., 2015), nasal sprays (de Pauw et al., 2017), and mouth rinses (Ataide-Silva et al., 2016) can also be used as carbohydrate intake forms. In summary, the lack of a positive finding regarding the role of CHO ingestion on EF may be related to the deficiencies in the experimental design in terms of intervention measures, such as insufficient intake of carbohydrates, a single type of carbohydrate intake, or the form of the CHO. For the analyses based on athletes’ programs were conducted and explored below.

#### 4.1.2. Subgroup analysis according to types of athletes

Sports can be divided into open skill sports and closed skill sports (Dai et al., 2013). Open skill sports are performed in a dynamic and changing environment, while closed skill sports are performed in a predictable and static environment (Honeybourne, 2006). In the studies we included, athletes in two studies were involved in closed skill sports in the long term (Hogervorst et al., 1999; Pollow et al., 2016) and in six studies they were involved in open skill sports (Winnick et al., 2005; Pomportes et al., 2016; Harper et al., 2017; Pomportes et al., 2019; Sun et al., 2020; Zhu et al., 2020). We performed a subgroup analysis based on this feature and the outcomes of RT were opposite (open skill sports: −0.27 [−0.60, 0.07]; closed skill sports: 0.29 [−0.24, 0.82]); however, the results of ACC remained insignificant for athletes with open skill sports (open skill sports: −0.10 [−0.34, 0.14]). The results showed that the intake of carbohydrate solutions was more beneficial for RT of athletes in open skill sports.

Possible reasons for this finding include early studies showing that carbohydrate intake can be accompanied by an increase in sympathetic nervous system (SNS) activity in animals and humans (Young and Landsberg, 1977; Welle, 1995). Also, different exercise patterns over time can result in different outcomes. In one of the studies included carbohydrate intake produced a very significant effect on post-exercise RT (Pomportes et al., 2019), in subjects who were elite pentathlon athletes. Modern pentathlon consists of five events that take one day to complete, including shooting, fencing, swimming, horseback riding, and trail running (Bertollo et al., 2009), for which fencing has higher requirements on reaction time. For fencers, the ability to respond quickly to specific fencing stimuli increased with training experience, and this difference was exacerbated by an increase in the number of stimulus-response options (Milic et al., 2020). It had also been shown that high-level shooters have better EF than novices, and they have better RT and ACC than novices in cognitive function tests, mainly due to strong resistance to interference (Shao et al., 2020). Previously, few researchers have focused on linking the effects of carbohydrate intake on EF in athletes to the needs of athletes in different sports, which indicates us that more research data are needed to explore this issue in the future.

In summary, the opposite results of the two sports types might be due to the fact that cognitive function was involved differently in different types of sports disciplines, and athletes who engaged in open skilled sports have higher cognitive function (Pačesová et al., 2020). For the subfield of EF, a study of 75 elite athletes found that time spent in different types of sports affects EF in elite athletes, and that cognitively demanding sports experiences may be beneficial for the development of EF (Koch and Krenn, 2021). Therefore, future research should appropriately focus more on athletes in programs that require more cognitive function to be involved. When looking at cognitive function, studies should narrow it down to sub-domains and even smaller level units. For interventions, we can focus on the type of carbohydrate, dose, form, and timing of administration.

### 4.2. Limitations

There are still deficiencies in this study. First, because we limited the outcome indicators to EF, this resulted in a small number of studies that met the criteria. Second, due to the included experiments containing crossover experiments, if the data from the experimental and control groups were processed directly in the same way as the parallel experiments, the confidence intervals would be wide and the weights of the experiments would be small (Higgins et al., 2022). For quality assessment, since it is a very subjective process, the evaluation results could vary from person to person. For the research level, there were no experiments in our included articles measured the subdomain of cognitive flexibility. After we analyzed the results, we found that specific areas of cognitive function are more important for athletes in certain specific sports, so in future studies, we should select the appropriate domains of cognitive function for the role of carbohydrates depending on the sports.

## 5. Conclusion

Overall, the intake of 6–12% of single or mixed carbohydrates drinks before or during exercise was not significantly effective in reducing the decline in EF after exercise. However, due to the small number of included studies, findings from additional RCTs need to be interpreted. Based on current evidence, our analysis of this result might be due to the different needs of the included subjects for cognitive function. Since, as a predictor of performance, motor cognitive function is likely to be an effective tool for national sports teams to improve their competitiveness (Yongtawee et al., 2022), we should pay attention to it. But currently, studies on higher levels of cognitive function, such as EF (Voss et al., 2010), in athletes are inadequate. In future studies, we can narrow the scope of cognitive function and investigate the effects of different doses, types, and forms of carbohydrate administration on a specific cognitive domain based on athletes’ sports.

## Data availability statement

The original contributions presented in the study are included in the article/Supplementary material, further inquiries can be directed to the corresponding author.

## Author contributions

JY, QH, and QW: conceptualization and validation. JY and QH: methodology and formal analysis. JY and QL: software. JY: writing—original draft preparation. QH, YS, XS, and QW: writing—review and editing. TL: visualization. QW: supervision and funding acquisition. JY, QH, QL, TL, YS, XS, and QW: project administration. All authors contributed to the article and approved the submitted version.

## Funding

This research was funded by the National Key R&D Program of China (2019YFF0301702) and BYHEALTH Co., Ltd. (C01202208315780).

## Conflict of interest

The authors declare that the research was conducted in the absence of any commercial or financial relationships that could be construed as a potential conflict of interest.

## Publisher’s note

All claims expressed in this article are solely those of the authors and do not necessarily represent those of their affiliated organizations, or those of the publisher, the editors and the reviewers. Any product that may be evaluated in this article, or claim that may be made by its manufacturer, is not guaranteed or endorsed by the publisher.
