# Supplementary material for: Effects of carbohydrate drinks ingestion on executive function in athletes: a systematic review and meta-analysis
Source: Front Psychol. 2023 Aug 10;14:1183460. doi: 10.3389/fpsyg.2023.1183460 (PMC10448191; doi:10.3389/fpsyg.2023.1183460)
Supplement: Supplementary file 1 [file Data_Sheet_1.ZIP › Supplementary Materials/File 1.docx]

WOS:

TS=(Central Nervous System OR Central Nervous Systems OR Nervous System, Central OR Nervous Systems, Central OR Systems, Central Nervous OR Cerebrospinal Axis OR Axi, Cerebrospinal OR Axis, Cerebrospinal OR Cerebrospinal Axi OR cognitoin OR Cognitions or Cognitive Function or Cognitive Functions or Function, Cognitive or Functions, Cognitive)

TS=(Athletes OR Athlete OR Professional Athletes OR Athlete, Professional OR Athletes, Professional OR Professional Athlete OR Elite Athletes OR Athlete, Elite OR Athletes, Elite OR Elite Athlete OR College Athletes OR Athlete, College OR Athletes, College OR College Athlete OR sports OR Sport OR Athletics OR Athletic OR Exercise OR Exercises OR Physical Activity OR Activities, Physical OR Activity, Physical OR Physical Activities OR Exercise, Physical OR Exercises, Physical OR Physical Exercise OR Physical Exercises OR Acute Exercise OR Acute Exercises OR Exercise, Acute OR Exercises, Acute OR Exercise, Isometric OR Exercises, Isometric OR Isometric Exercises OR Isometric Exercise OR Exercise, Aerobic OR Aerobic Exercise OR Aerobic Exercises OR Exercises, Aerobic OR Exercise Training OR Exercise Trainings OR Training, Exercise OR Trainings, Exercise)

TS=(Sugars OR Sugar OR Carbohydrates OR Carbohydrate OR Glucose OR D-Glucose OR D Glucose OR Dextrose OR Glucose, (alpha-D)-Isomer OR Anhydrous Dextrose OR Dextrose, Anhydrous OR Glucose, (DL)-Isomer OR Glucose, (L)-Isomer OR L-Glucose OR L Glucose OR Glucose Monohydrate OR Monohydrate, Glucose OR Glucose, (beta-D)-Isomer OR Beverages OR Beverage OR Sugar-Sweetened Beverages OR Beverage, Sugar-Sweetened OR Beverages, Sugar-Sweetened OR Sugar-Sweetened Beverage OR Sugar-Added Beverage OR Beverage, Sugar-Added OR Beverages, Sugar-Added OR Sugar Added Beverage OR Sugar-Added Beverages OR Sugar Added Beverages OR Sugar Sweetened Beverage OR Beverage, Sugar Sweetened OR Beverages, Sugar Sweetened OR Sugar Sweetened Beverages OR Sweetened Beverage, Sugar OR Sweetened Beverages, Sugar OR Sweetened Drinks OR Sweetened Drink OR Drink, Sweetened OR Drinks, Sweetened OR Sugar-Sweetened Soft Drinks OR Sugar Sweetened Soft Drinks OR Sugar-Sweetened Soft Drink OR Drink, Sugar-Sweetened Soft OR Drinks, Sugar-Sweetened Soft OR Soft Drink, Sugar-Sweetened OR Soft Drinks, Sugar-Sweetened OR Sugar Sweetened Soft Drink OR Sweetened Beverages OR Sweetened Beverage OR Beverage, Sweetened OR Beverages, Sweetened OR Sugar-Sweetened Sodas OR Sugar Sweetened Sodas OR Sugar-Sweetened Soda OR Soda, Sugar-Sweetened OR Sodas, Sugar-Sweetened OR Sugar Sweetened Soda)

The keywords were combined with **AND.**

PubMed:

(((("Central Nervous System"[Mesh] OR Central Nervous Systems OR Nervous System, Central OR Nervous Systems, Central OR Systems, Central Nervous OR Cerebrospinal Axis OR Axi, Cerebrospinal OR Axis, Cerebrospinal OR Cerebrospinal Axi) OR ("Cognition"[Mesh] OR Cognitions OR Cognitive Function OR Cognitive Functions OR Function, Cognitive OR Functions, Cognitive)) AND ((((("Sugar-Sweetened Beverages"[Mesh] OR Beverage, Sugar-Sweetened OR Beverages, Sugar-Sweetened OR Sugar-Sweetened Beverage OR Sugar-Added Beverage OR Beverage, Sugar-Added OR Beverages, Sugar-Added OR Sugar Added Beverage OR Sugar-Added Beverages OR Sugar Added Beverages OR Sugar Sweetened Beverage OR Beverage, Sugar Sweetened OR Beverages, Sugar Sweetened OR Sugar Sweetened Beverages OR Sweetened Beverage, Sugar OR Sweetened Beverages, Sugar OR Sweetened Drinks OR Sweetened Drink OR Drink, Sweetened OR Drinks, Sweetened OR Sugar-Sweetened Soft Drinks OR Sugar Sweetened Soft Drinks OR Sugar-Sweetened Soft Drink OR Drink, Sugar-Sweetened Soft OR Drinks, Sugar-Sweetened Soft OR Soft Drink, Sugar-Sweetened OR Soft Drinks, Sugar-Sweetened OR Sugar Sweetened Soft Drink OR Sweetened Beverages OR Sweetened Beverage OR Beverage, Sweetened OR Beverages, Sweetened OR Sugar-Sweetened Sodas OR Sugar Sweetened Sodas OR Sugar-Sweetened Soda OR Soda, Sugar-Sweetened OR Sodas, Sugar-Sweetened OR Sugar Sweetened Soda) OR ("Beverages"[Mesh] OR Beverage)) OR ("Sugars"[Mesh] OR Sugar)) OR ("Glucose"[Mesh] OR D-Glucose OR D Glucose OR Dextrose OR Glucose, (alpha-D)-Isomer OR Anhydrous Dextrose OR Dextrose, Anhydrous OR Glucose, (DL)-Isomer OR Glucose, (L)-Isomer OR L-Glucose OR L Glucose OR Glucose Monohydrate OR Monohydrate, Glucose OR Glucose, (beta-D)-Isomer)) OR ("Carbohydrates"[Mesh] OR Carbohydrate)))) AND (((("Exercise"[Mesh]) OR (((((((((((((((((((((((((Exercises[Title/Abstract]) OR (Physical Activity[Title/Abstract])) OR (Activities, Physical[Title/Abstract])) OR (Activity, Physical[Title/Abstract])) OR (Physical Activities[Title/Abstract])) OR (Exercise, Physical[Title/Abstract])) OR (Exercises, Physical[Title/Abstract])) OR (Physical Exercise[Title/Abstract])) OR (Physical Exercises[Title/Abstract])) OR (Acute Exercise[Title/Abstract])) OR (Acute Exercises[Title/Abstract])) OR (Exercise, Acute[Title/Abstract])) OR (Exercises, Acute[Title/Abstract])) OR (Exercise, Isometric[Title/Abstract])) OR (Exercises, Isometric[Title/Abstract])) OR (Isometric Exercises[Title/Abstract])) OR (Isometric Exercise[Title/Abstract])) OR (Exercise, Aerobic[Title/Abstract])) OR (Aerobic Exercise[Title/Abstract])) OR (Aerobic Exercises[Title/Abstract])) OR (Exercises, Aerobic[Title/Abstract])) OR (Exercise Training[Title/Abstract])) OR (Exercise Trainings[Title/Abstract])) OR (Training, Exercise[Title/Abstract])) OR (Trainings, Exercise[Title/Abstract]))) OR (("Sports"[Mesh]) OR (((Sport[Title/Abstract]) OR (Athletics[Title/Abstract])) OR (Athletic[Title/Abstract])))) OR (("Athletes"[Mesh]) OR (((((((((((((Athlete[Title/Abstract]) OR (Professional Athletes[Title/Abstract])) OR (Athlete, Professional[Title/Abstract])) OR (Athletes, Professional[Title/Abstract])) OR (Professional Athlete[Title/Abstract])) OR (Elite Athletes[Title/Abstract])) OR (Athlete, Elite[Title/Abstract])) OR (Athletes, Elite[Title/Abstract])) OR (Elite Athlete[Title/Abstract])) OR (College Athletes[Title/Abstract])) OR (Athlete, College[Title/Abstract])) OR (Athletes, College[Title/Abstract])) OR (College Athlete[Title/Abstract]))))

SPORTDiscus:

AB ( Cognition OR Cognitions OR Cognitive Function OR Cognitive Functions OR Function, Cognitive OR Functions, Cognitive OR Central Nervous System OR Central Nervous Systems OR Nervous System, Central OR Nervous Systems, Central OR Systems, Central Nervous OR Cerebrospinal Axis OR Axi, Cerebrospinal OR Axis, Cerebrospinal OR Cerebrospinal Axi ) AND AB ( Athletes OR Athlete OR Professional Athletes OR Athlete, Professional OR Athletes, Professional OR Professional Athlete OR Elite Athletes OR Athlete, Elite OR Athletes, Elite OR Elite Athlete OR College Athletes OR Athlete, College OR Athletes, College OR College Athlete OR Exercises OR Physical Activity OR Activities, Physical OR Activity, Physical OR Physical Activities OR Exercise, Physical OR Exercises, Physical OR Physical Exercise OR Physical Exercises OR Acute Exercise OR Acute Exercises OR Exercise, Acute OR Exercises, Acute OR Exercise, Isometric OR Exercises, Isometric OR Isometric Exercises OR Isometric Exercise OR Exercise, Aerobic OR Aerobic Exercise OR Aerobic Exercises OR Exercises, Aerobic OR Exercise Training OR Exercise Trainings OR Training, Exercise OR Trainings, Exercise OR Sports OR sport OR Athletics OR Athletic ) AND AB ( Sugars OR sugar OR carbohydrates OR carbohydrate OR glucose OR D Glucose OR D Glucose OR Dextrose OR Glucose, (alpha D) Isomer OR Anhydrous Dextrose OR Dextrose, Anhydrous OR Glucose, (DL) Isomer OR Glucose, (L) Isomer OR L Glucose OR Glucose Monohydrate OR Monohydrate, Glucose OR Glucose, (beta D) Isomer OR Beverages OR Beverage OR Sugar Sweetened Beverages OR Beverage, Sugar Sweetened OR Beverages, Sugar Sweetened OR Sugar Sweetened Beverage OR Sugar Added Beverage OR Beverage, Sugar Added OR Beverages, Sugar Added OR Sugar Added Beverage OR Sugar Added Beverages OR Sugar Added Beverages OR Sugar Sweetened Beverage OR Beverage, Sugar Sweetened OR Beverages, Sugar Sweetened OR Sugar Sweetened Beverages OR Sweetened Beverage, Sugar OR Sweetened Beverages, Sugar OR Sweetened Drinks OR Sweetened Drink OR Drink, Sweetened OR Drinks, Sweetened OR Sugar Sweetened Soft Drinks OR Sugar Sweetened Soft Drinks OR Sugar Sweetened Soft Drink OR Drink, Sugar Sweetened Soft OR Drinks, Sugar Sweetened Soft OR Soft Drink, Sugar Sweetened OR Soft Drinks, Sugar Sweetened OR Sugar Sweetened Soft Drink OR Sweetened Beverages OR Sweetened Beverage OR Beverage, Sweetened OR Beverages, Sweetened OR Sugar Sweetened Sodas OR Sugar Sweetened Sodas OR Sugar Sweetened Soda OR Soda, Sugar Sweetened OR Sodas, Sugar Sweetened OR Sugar Sweetened Soda )

Cochrane:


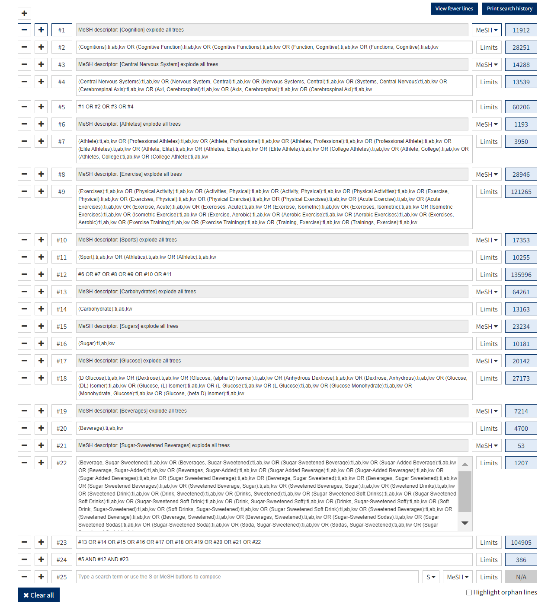


Embase:

'Cognitions':ab,ti OR 'Cognitive Function':ab,ti OR 'Cognitive Functions':ab,ti OR 'Function, Cognitive':ab,ti OR 'Functions, Cognitive':ab,ti OR 'Cognition':ab,ti OR 'Central Nervous System':ab,ti OR 'Central Nervous Systems':ab,ti OR 'Nervous System, Central':ab,ti OR 'Nervous Systems, Central':ab,ti OR 'Systems, Central Nervous':ab,ti OR 'Cerebrospinal Axis':ab,ti OR 'Axi, Cerebrospinal':ab,ti OR 'Axis, Cerebrospinal':ab,ti OR 'Cerebrospinal Axi':ab,ti AND 'sugars':ab,ti OR 'sugar':ab,ti OR 'carbohydrate':ab,ti OR 'carbohydrates':ab,ti OR 'glucose':ab,ti OR 'D Glucose':ab,ti OR 'Dextrose':ab,ti OR 'Glucose, (alpha D) Isomer':ab,ti OR 'Anhydrous Dextrose':ab,ti OR 'Dextrose, Anhydrous':ab,ti OR 'Glucose, (DL) Isomer':ab,ti OR 'Glucose, (L) Isomer':ab,ti OR 'L Glucose':ab,ti OR 'Glucose Monohydrate':ab,ti OR 'Monohydrate, Glucose':ab,ti OR 'Glucose, (beta D) Isomer':ab,ti OR 'beverage':ab,ti OR 'beverages':ab,ti OR 'Sugar Sweetened Beverages':ab,ti OR 'Beverage, Sugar Sweetened':ab,ti OR 'Beverages, Sugar Sweetened':ab,ti OR 'Suga Sweetened Beverage':ab,ti OR 'Sugar Added Beverage':ab,ti OR 'Beverage, Sugar Added':ab,ti OR 'Beverages, Sugar Added':ab,ti OR 'Sugar Added Beverage':ab,ti OR 'Sugar Added Beverages':ab,ti OR 'Sugar Added Beverages':ab,ti OR 'Sugar Sweetened Beverage':ab,ti OR 'Beverage, Sugar Sweetened':ab,ti OR 'Beverages, Sugar Sweetened':ab,ti OR 'Sugar Sweetened Beverages':ab,ti OR 'Sweetened Beverage, Sugar':ab,ti OR 'Sweetened Beverages, Sugar':ab,ti OR 'Sweetened Drinks':ab,ti OR 'Sweetened Drink':ab,ti OR 'Drink, Sweetened':ab,ti OR 'Drinks, Sweetened':ab,ti OR 'Sugar Sweetened Soft Drinks':ab,ti OR 'Sugar Sweetened Soft Drinks':ab,ti OR 'Sugar Sweetened Soft Drink':ab,ti OR 'Drink, Sugar Sweetened Soft':ab,ti OR 'Drinks, Sugar Sweetened Soft':ab,ti OR 'Soft Drink, Sugar Sweetened':ab,ti OR 'Soft Drinks, Sugar Sweetened':ab,ti OR 'Sugar Sweetened Soft Drink':ab,ti OR 'Sweetened Beverages':ab,ti OR 'Sweetened Beverage':ab,ti OR 'Beverage, Sweetened':ab,ti OR 'Beverages, Sweetened':ab,ti OR 'Sugar Sweetened Sodas':ab,ti OR 'Sugar Sweetened Sodas':ab,ti OR 'Sugar Sweetened Soda':ab,ti OR 'Soda, Sugar Sweetened':ab,ti OR 'Sodas, Sugar Sweetened':ab,ti OR 'Sugar Sweetened Soda':ab,ti AND 'sport':ab,ti OR 'sports':ab,ti OR 'Athletics':ab,ti OR 'Athletic':ab,ti OR 'Athletes':ab,ti OR 'Athlete':ab,ti OR 'Professional Athletes':ab,ti OR 'Athlete, Professional':ab,ti OR 'Athletes, Professional':ab,ti OR 'Professional Athlete':ab,ti OR 'Elite Athletes':ab,ti OR 'Athlete, Elite':ab,ti OR 'Athletes, Elite':ab,ti OR 'Elite Athlete':ab,ti OR 'College Athletes':ab,ti OR 'Athlete, College':ab,ti OR 'Athletes, College':ab,ti OR 'College Athlete':ab,ti OR 'exercise':ab,ti OR 'Exercises':ab,ti OR 'Physical Activity':ab,ti OR 'Activities, Physical':ab,ti OR 'Activity, Physical':ab,ti OR 'Physical Activities':ab,ti OR 'Exercise, Physical':ab,ti OR 'Exercises, Physical':ab,ti OR 'Physical Exercise':ab,ti OR 'Physical Exercises':ab,ti OR 'Acute Exercise':ab,ti OR 'Acute Exercises':ab,ti OR 'Exercise, Acute':ab,ti OR 'Exercises, Acute':ab,ti OR 'Exercise, Isometric':ab,ti OR 'Exercises, Isometric':ab,ti OR 'Isometric Exercises':ab,ti OR 'Isometric Exercise':ab,ti OR 'Exercise, Aerobic':ab,ti OR 'Aerobic Exercise':ab,ti OR 'Aerobic Exercises':ab,ti OR 'Exercises, Aerobic':ab,ti OR 'Exercise Training':ab,ti OR 'Exercise Trainings':ab,ti OR 'Training, Exercise':ab,ti OR 'Trainings, Exercise':ab,ti

Scopus:

((TITLE-ABS-KEY("Sugar-Sweetened Beverages" OR "Beverage, Sugar-Sweetened" OR "Beverages, Sugar-Sweetened" OR "Sugar-Sweetened Beverage" OR "Sugar-Added Beverage" OR "Beverage, Sugar-Added" OR "Beverages, Sugar-Added" OR "Sugar Added Beverage" OR "Sugar-Added Beverages" OR "Sugar Added Beverages" OR "Sugar Sweetened Beverage" OR "Beverage, Sugar Sweetened" OR "Beverages, Sugar Sweetened" OR "Sugar Sweetened Beverages" OR "Sweetened Beverage, Sugar" OR "Sweetened Beverages, Sugar" OR "Sweetened Drinks" OR "Sweetened Drink" OR "Drink, Sweetened" OR "Drinks, Sweetened" OR "Sugar-Sweetened Soft Drinks" OR "Sugar Sweetened Soft Drinks" OR "Sugar-Sweetened Soft Drink" OR "Drink, Sugar-Sweetened Soft" OR "Drinks, Sugar-Sweetened Soft" OR "Soft Drink, Sugar-Sweetened" OR "Soft Drinks, Sugar-Sweetened" OR "Sugar Sweetened Soft Drink" OR "Sweetened Beverages" OR "Sweetened Beverage" OR "Beverage, Sweetened" OR "Beverages, Sweetened" OR "Sugar-Sweetened Sodas" OR "Sugar Sweetened Sodas" OR "Sugar-Sweetened Soda" OR "Soda, Sugar-Sweetened" OR "Sodas, Sugar-Sweetened" OR "Sugar Sweetened Soda" OR "Carbohydrates" OR "Carbohydrate" OR "Sugars" OR "Sugar")) OR (TITLE-ABS-KEY("Glucose" OR "D-Glucose" OR "D Glucose" OR "Dextrose" OR "Glucose, (alpha-D)-Isomer" OR "Anhydrous Dextrose" OR "Dextrose, Anhydrous" OR "Glucose, (DL)-Isomer" OR "Glucose, (L)-Isomer" OR "L-Glucose" OR "L Glucose" OR "Glucose Monohydrate" OR "Monohydrate, Glucose" OR "Glucose, (beta-D)-Isomer"))) AND ((TITLE-ABS-KEY("Sports" OR "Sport" OR "Athletics" OR "Athletic")) OR (TITLE-ABS-KEY("Exercise" OR "Exercises" OR "Physical Activity" OR "Activities, Physical" OR "Activity, Physical" OR "Physical Activities" OR "Exercise, Physical" OR "Exercises, Physical" OR "Physical Exercise" OR "Physical Exercises" OR "Acute Exercise" OR "Acute Exercises" OR "Exercise, Acute" OR "Exercises, Acute" OR "Exercise, Isometric" OR "Exercises, Isometric" OR "Isometric Exercises" OR "Isometric Exercise" OR "Exercise, Aerobic" OR "Aerobic Exercise" OR "Aerobic Exercises" OR "Exercises, Aerobic" OR "Exercise Training" OR "Exercise Trainings" OR "Training, Exercise" OR "Trainings, Exercise")) OR (TITLE-ABS-KEY("Athletes" OR "Athlete" OR "Professional Athletes" OR "Athlete, Professional" OR "Athletes, Professional" OR "Professional Athlete" OR "Elite Athletes" OR "Athlete, Elite" OR "Athletes, Elite" OR "Elite Athlete" OR "College Athletes" OR "Athlete, College" OR "Athletes, College" OR "College Athlete"))) AND ((TITLE-ABS-KEY("Cognition" OR "Cognitions" OR "Cognitive Function" OR "Cognitive Functions" OR "Function, Cognitive" OR "Functions, Cognitive")) OR (TITLE-ABS-KEY("Central Nervous System" OR "Central Nervous Systems" OR "Nervous System, Central" OR "Nervous Systems, Central" OR "Systems, Central Nervous" OR "Cerebrospinal Axis" OR "Axi, Cerebrospinal" OR "Axis, Cerebrospinal" OR "Cerebrospinal Axi")))
